# Supplementary material for: Splice-Junction-Based Mapping of Alternative Isoforms in the Human Proteome
Source: Cell Rep. Author manuscript; Available in PMC 2020 Jan 15. (PMC6961840; doi:10.1016/j.celrep.2019.11.026)

A

sp|O14618|CCS_HUMAN|ENSG00000173992|RI1|1825|chr11|66599253|66599636|+2|r31|T4
GSGQLRVQDVEVHLEDQM[15.99]VLVHTTLPSQEVQALLEGTGR q value: 4.2091e−05 Tr_novel:TRUE RefSeq_Nove
Search result spec prec mz: 1072.3095 Actual spec prec mz: 1072.3096
Fragments matched per AA: 1.38 Proportion of top 20 peaks matched: 0.4

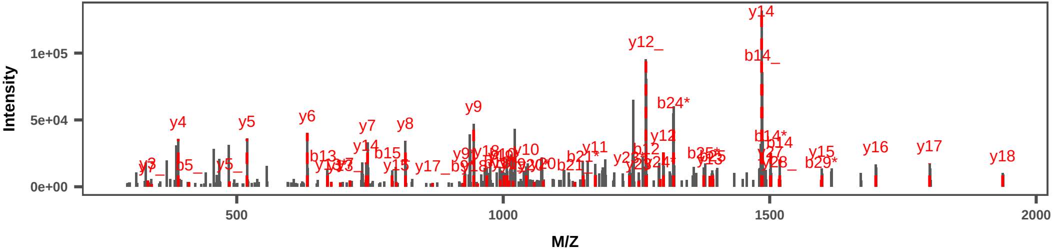

B

Scatterplot of predicted elution time
Fitting R2: 0.853
Novel peptide residual Z score: 0.951
Number of peptides: 1792

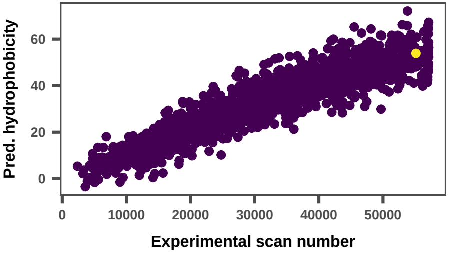

C

Distributions of residuals from best−fit line
of predicted RT vs Expt. scan number
Line: Z score of novel peptide
Z: 0.951

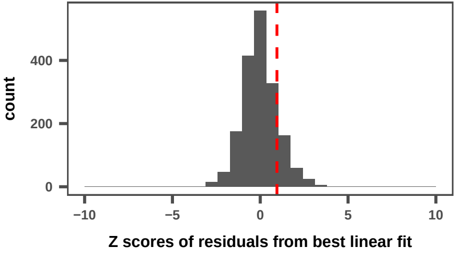

Supplement: 2 [file NIHMS1546469-supplement-2.zip › DF1/PXD010154/AdrenalGland/AdrenalGland_5_CCS_GSGQLRVQDVEVHLEDQMVLVHTTLPSQEVQALLEGTGR.pdf]
